# Immunotherapy in Chronic Lymphocytic Leukaemia (CLL)

**DOI:** 10.1007/s11899-015-0295-9

**Published:** 2016-02-09

**Authors:** Ciara L. Freeman, John G. Gribben

**Affiliations:** John Vane Cancer Centre, Charterhouse Square, Barts Cancer Institute, Queen Mary University of London, London, UK

**Keywords:** Lymphoid neoplasia, Chronic lymphocytic leukaemia (CLL), Immunotherapy, Monoclonal antibody, Immune checkpoint inhibitors, Immunomodulatory drugs

## Abstract

Chronic lymphocytic leukaemia (CLL) is well known to generate impaired immune responses in the host, with the malignant clone residing in well-vascularized tissues and circulating in peripheral blood but also in close proximity to effector cells that are capable, if activated appropriately, of eliciting a cytotoxic response. These, combined with the fact that this is frequently a condition affecting older patients with co-morbidities often unfit for many “traditional” cytotoxic agents with their significant associated toxicities, make CLL an ideal candidate for the development of immunotherapy. The impressive results seen with the addition of a monoclonal antibody, rituximab, to a chemotherapy backbone, for example, is testament to how effective harnessing an immune-mediated response in CLL can be. This review serves to outline the available arsenal of immunotherapies—past and present—demonstrated to have potential in CLL with some perspectives on how the landscape in this disease may evolve in the future.

## Introduction

Cancer immunotherapy, described by the National Cancer Institute (NCI) as any “biological therapy that uses substances to stimulate or suppress the immune system to help the body fight cancer…”, has been the subject of intense scientific interest over the past three decades [1]. Many acknowledge that hematologic malignancies, with cells that are readily isolated and manipulated, developing in close proximity to sites of or arising from cells involved with immune response and recognition, have paved the way for understanding and innovation in this field [2••].

Treatment options for patients with chronic lymphocytic leukaemia (CLL) have evolved over time, from alkylator-based chlorambucil or cyclophosphamide in the 1970s to combinations with purine analogues in the 1990s [3, 4]. Similar to other cancers, a therapeutic ceiling was reached with addition of further traditional “chemotherapy” not translating into improvements in overall responses (OR) or survival (OS) [3]. The introduction of immunotherapy (in the form of a monoclonal antibody targeting CD20, rituximab) to a chemotherapeutic backbone demonstrated significant improvements in OR and OS rates in the front line setting [5] and established its role in the treatment paradigm.

For today’s patients, the future has never been brighter and recent therapeutic improvements in CLL were labelled by the American Society of Clinical Oncology (ASCO) as “the Cancer advance of the year” in its annual report in 2015 [6]. In the current era, it is highly unlikely that a newly diagnosed patient with CLL will not be treated with some form of immunotherapy during the course of their disease [7••], and the repertoire of available immunotherapies to treat CLL is likely to increase significantly over the coming years.

Although traditionally subdivided into “passive” or “active” based on the ability to engage an immune response against malignant cells in the host, this may not be an entirely accurate division [8]. Many passive immunotherapies will illicit cytokine release, generate tumour associated antigens which will be taken up by antigen-presenting cells (APCs) or require native immune cells to effect cell death [9•]. This review will detail the scope of agents with the ability to generate an immune response and potential utility in CLL, including those with an established role (e.g., anti-CD20 monoclonal antibodies/mAb) to novel strategies such as checkpoint inhibitors and cellular therapies.

## Monoclonal Antibodies

**Fig. 1:**
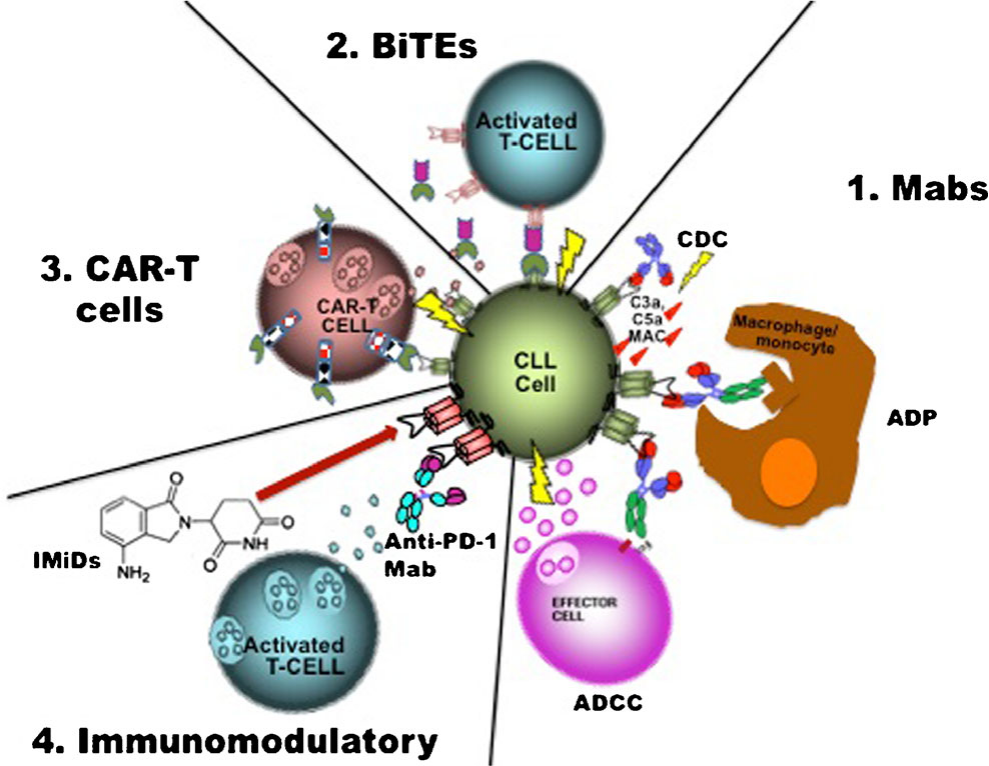
Mechanism of action of immunotherapies available in CLL. *1*. Monoclonal antibodies (*Mabs*) act via several mechanisms to recruit an immune response, targeting a tumour-specific antigen (TSA) and generating to varying degrees depending on the antibody: complement activation (*CDC*), activation of cytotoxic effector cells via the Fc gamma receptor (*ADCC*) or activating phagocytosis (*ADP*). *2*. Bi-specific T cell engaging antibodies activate T cells in close proximity to the malignant clone—one portion is specific for the TSA on the clone and will only bind to the CD3 receptor on the T cells when the TSA fragment is bound, thus limiting the T cell response to sites of disease. *3*. Adaptive T cell transfer with chimeric antigen receptor T cells allows for the re-infusion of autologous T cells primed to recognize a TSA that will generate a T cell response upon binding due to the co-stimulatory domains that are built into the receptor complex. *4*. Blockade or downregulation of PD-1 by either a monoclonal antibody or through the action of immunomodulatory agents like lenolidomide overcomes the inhibition of T cells and generates an immune response against the malignant clone. *Mab* monoclonal antibody, *CDC* complement dependent cytotoxicity, *ADCC* antibody dependent cellular cytotoxicity, *ADP* antibody dependent phagocytosis, *MAC* membrane attack complex, *BiTE* bi-specific T cell engaging antibody, *CAR* chimeric antigen receptor, *IMiD* immunomodulatory drug, *PD*-*1* programmed cell death-1

Antibodies cloned to target a tumour-specific antigen (TSA) are possibly the best characterized and most extensively employed immunotherapy currently in CLL. Numerous targets exist to selectively target B cells—such as CD20, CD19 and CD37. Composed of a fixed effector cell binding region (Fc) and a variable region with specificity for the TSA, these large molecules act to recruit an immune response predominantly by opsonising cancer cells, flagging them for destruction by effector cells via antibody dependent cell-mediated cytotoxicity (ADCC) and antibody-dependent cellular phagocytosis (ADP) [3]. Recruitment of the complement cascade or complement-dependent cytotoxicity (CDC) to varying degrees also plays a role in the cell death initiated by these agents [10, 11]—Fig. 1. Advances in technology have led to the development of fully humanized and glycoengineered antibodies with even greater specificity for the TSA and enhanced recruitment of the immune response with associated improvements in clinical efficacy [12–15].

## Anti-CD20 Monoclonal Antibodies (Rituximab, Ofatumumab, Obinutuzumab)

CD20 is a hydrophobic glycosylated transmembrane protein present on the cell surface of mature B lymphocytes [16] but not stem cells, pro-B cells or plasma cells [17]. It has no natural ligand [18], and although both CD19-induced calcium responses and B cell receptor signaling is altered in CD20 knockout mice [19], its exact function remains poorly elucidated. However, it appears to be neither shed nor internalized [20], and its specificity for B cells makes it the perfect target to treat B cell neoplasms.

The first approved therapeutic antibody for the treatment of malignancy [3], rituximab, is an IgG1 κ chimeric immunoglobulin containing both murine light- and heavy-chain variable region sequences with human constant region sequences. It is thought to exert its cytotoxic effects on CD20-expressing B cells chiefly by ADCC, ADP and to a lesser extent via CDC [10, 11].

Although it had limited success as a single agent [21], further investigation into its use in CLL proved its efficacy across a variety of combination strategies in phase II and III clinical trials and cemented its place in the treatment of both treatment-naive and relapsed patients [5, 7••, 22, 23]. It remains a crucial component of the “gold standard” for patients with CLL who are deemed “fit” for full-dose fludarabine, as part of the Fludarabine Cyclophosphamide Rituximab (FCR) regime, and this strategy has yet to be outperformed in terms of OR and OS rates in the frontline setting, although it is not suitable for patients who have a 17p deletion or TP53 mutation [7••].

Ofatumumab is a fully humanized mAb that targets a different epitope on the CD20 molecule than rituximab and has a slower dissociation rate, greater CDC and similar ADCC properties [24]. Although it was granted approval in the treatment of fludarabine- and alemtuzumab-refractory patients by the EMA and the FDA [7••, 25], its uptake as a single agent in this patient population has now been limited given the inferior activity of this agent in comparison with ibrutinib, also in a randomized clinical trial that led to regulatory approval of this Bruton’s tyrosine kinase (BTK) inhibitor [26]. Ofatumumab has also been granted FDA approval for the treatment of therapy-naïve “unfit” patients, i.e., those deemed unsuitable for full-dose fludarabine-based therapy in the upfront setting when given in combination with oral chlorambucil. Compared to chlorambucil monotherapy, the combination offered a significant improvement in OR rates (82 vs 69 %) and progression-free survival (PFS; 22.4 vs 13.1 months) [27].

Obinutuzumab is also fully humanized but is also glycoengineered to reduce the fucose content of the Fc portion, to enhance the binding of the Fcgamma receptor on effector cells and increase cytotoxic potency via ADCC and ADP mechanisms [12–14, 28, 29]. When directly compared to either chlorambucil monotherapy or a combination of chlorambucil and rituximab (R-CLB), the combination of obinutuzimab and chlorambucil (G-CLB) outperformed both treatment arms in unfit previously untreated patients and the head-to-head comparison between G-CLB and R-CLB achieved a statistically significant improvement of PFS (29.2 vs 15.4 months, respectively) with a significantly higher number of complete responses in the G-CLB group (20.7 vs 7.0 %) [15, 30]. Whether these results will translate into a greater tendency to use this regime over ofatumumab with chlorambucil remains to be seen, but both regimens have approval in the frontline setting for this unfit patient population [25]. Further clinical trials comparing obinutuzumab using alternative combinations are ongoing, and whether it will continue to outperform rituximab in other settings remains to be seen.

## Anti-CD52 (Alemtuzumab)

Alemtuzumab is a fully humanized IgG1 monoclonal antibody directed against the heavily glycosylated transmembrane glycoprotein, CD52. Unlike CD20, this antigen is not restricted to B cells and is expressed also by T lymphocytes, granulocytes, monocytes and macrophages as well as NK and dendritic cells [31]. Alemtuzumab exerts its cytotoxic activity primarily through CDC [31, 32] and ADCC [33] but has also been demonstrated to induce cell death via a direct mechanism that was independent of TP53 status [34], a finding which was later corroborated by clinical activity in this difficult-to-treat group with 17p deletion or TP53 mutations [35, 36]. It appears to have the greatest efficacy in those patients with greater circulating disease or bone marrow infiltration, with poorer responses in those patients with bulky lymph nodes (LN) (>5 cm in particular). Whether this is the result of poor penetration of this molecule into nodal tissue or the result of impaired recruitment of an immune response due to lower effector cell density in LNs is postulated but remains unproven [3]. Although it had approval to treat patients with CLL and continues to be described as a therapeutic option, a strategic decision by Sanofi has led to its withdrawal from the market for this indication and it can only be accessed via a compassionate use program [7••].

## Novel TSA Targets: Mabs Directed Against CD19, CD37, CD40

A transmembrane glycoprotein found on a wide range of B cell malignancies, CD19, is highly expressed in CLL and thus attractive TSA target for immunotherapies [37]. It acts as a co-stimulatory molecule for the B cell receptor, and development of mAbs was initially hampered by antigen internalization [38]. Improved technology has led to the development of a series of modified antibodies that re-instated its value as a potential target. MEDI-551, an afucosylated anti-CD19 antibody that acts predominantly via ADCC and demonstrated a 30 % response rate in CLL as a monotherapy, is under investigation in combination with bendamustine in a phase II setting in patients with relapsed/refractory (RR) CLL (NCT01466153). Preliminary analysis has reported clinical activity and comparable safety when compared to rituximab and bendamustine [39]. XmAb5574 (MOR00208) is another anti-CD19 mAb with an engineered Fc region to enhance ADCC and ADP [38]. Phase I evaluation as a monotherapy (NCT01161511) also demonstrated a 30 % response rate in RR CLL patients and acceptable toxicity. Its combination with lenolidomide is under investigation in a phase II setting (NCT02005289).

CD37 is another lineage-specific B cell TSA that is a prime candidate for targeted development. Otlertuzumab (TRU-016) is a novel small modular immunopharmaceutical (SMIP) that targets CD37 and demonstrates efficient ADCC and caspase-independent cytotoxicity that spares T cells, in contrast to alemtuzumab [40]. In a phase I dose escalation study (NCT00614042) involving patients with RR CLL, a 23 % response rate was seen and authors reported acceptable toxicity [41]. Further studies investigating its use in RR CLL patients in combination with bendamustine or anti-CD20 mAbs are ongoing [42].

CD40, a member of the tumour necrosis factor receptor superfamily, is expressed by 90–100 % of CLL cells [43], and its activation has been associated with enhanced survival of neoplastic B cells; triggering phosphorylation of ERK 1/2 and upregulating Mcl-1 and Bcl-xl and it may have a role in resistance to chemotherapy [44]. Lucatumumab (HCD122) is a humanized anti-CD40 antagonist that blocks the receptor from interacting with its natural ligand (CD40L) as well as mediating ADCC. In a phase I evaluation of patients with RR CLL, stable disease was observed in 17/26 patients with acceptable toxicity [45]. Despite the promise of initial pre-clinical work on another anti-CD40 mAb, dacetuzumab (SGN40), demonstrating ADCC that was further enhanced by combining it with lenolidomide [46], development beyond a phase I dose escalation study appears to have been halted in CLL after it demonstrated minimal clinical activity as a single agent [47].

## Bi-Specific T cell Engager (BiTE®): Blinatumomab

Blinatumomab is a recombinant fusion single-chain antibody with bi-specific properties, composed of an anti-CD3 fragment linked to an anti-CD19 fragment; this novel antibody has the ability to opsonise CD19+ cells and promote direct immune synapse formation with T cells [48]—Fig. 1. This novel construct, known as a bi-specific T cell engager (BiTE®), has already demonstrated impressive clinical efficacy in the treatment of relapsed or refractory acute lymphoblastic leukaemia and has received FDA approval for this indication [49]. Studies in indolent non-Hodgkin’s lymphoma and diffuse large B cell lymphoma are ongoing and provisional results are encouraging [50, 51]. Given the known T cell dysfunction in patients with CLL, there might be a theoretical obstacle against using this agent in this population [52•]. However, preliminary work demonstrated that the agent is active at least in vitro, in CLL samples with an “exhausted” T cell population [48]. Despite these findings, there appears to be no active plans, at least at the present, to explore the activity of this agent in vivo in a CLL population [42] which may have more to do with the competitive landscape in CLL than the probability of clinical efficacy.

## Immune Checkpoint Inhibitors: Anti-PD1/PD-L1 Antibodies

The discovery that malignant cells can evade the host immune system and its tumour surveillance mechanisms by inhibiting T cells has led to the development of a totally new class of immunotherapy—immune checkpoint inhibitors. Programmed cell death 1 (PD-1; CD279) and its ligands programmed death-ligand 1 (PD-L1; B7-H1; CD274) and PD-L2 (B7-DC; CD273) have been identified as possibly the most important axis in the maintenance of a malignant pro-survival microenvironment [53]. CLL cells have been shown to upregulate PD-L1 expression and suppress host T cell effector responses, exacerbating the development of an “exhausted” T cell phenotype, which overexpresses the PD-1 receptor and rendered incapable of attacking the malignant clone [52•, 54•, 55•]. Given the recent success using antibodies that can interfere with this immunosuppressive pathway in both Hodgkin’s [56] and non-Hodgkin’s lymphomas [57] and the weighty pre-clinical evidence referenced above, it seems clear that interference with this pathway should benefit patients with CLL. It seems unlikely that these antibodies would be developed as a monotherapy, but instead combined with other agents, for example, BTK inhibitors. Pre-clinical data have demonstrated synergy [58] with dual inhibition and a clinical trial (NCT02420912) involving relapsed, refractory or high-risk treatment-naive patients with CLL and the combination of ibrutinib and nivolumab (humanized IgG4 anti-PD-1 monoclonal antibody) is currently recruiting.

## Immunomodulatory Agent: Lenalidomide

The effects of lenalidomide, a second-generation thalidomide analogue and classified as an immunomodulatory drug (IMiD), have been increasingly well characterized over the past two decades [59]. Although IMiDs have some intrinsic anti-neoplastic activity, they are better considered as active immunotherapies [9•]. Lenalidomide has the ability to reverse the T cell dysfunction observed in patients with CLL in vitro, as well as to induce downregulation of PD-1 on these defective T cells and reduce PDL-1 expression by the malignant CLL clone [60•, 61]. These actions restore T cell effector function in addition to providing rationale for the combination of this agent with mAbs that interfere with the PD-1/PDL-1 axis.

Clinically, lenalidomide as a single agent has demonstrated responses in 56 % of previously untreated CLL patients [62] with encouraging activity in patients with high-risk cytogenetics such as del 17p [63]. Combination with rituximab (the so-called R-squared regimen) has been shown to further improve upon these responses, with OR rates of 83 % including in those with unmutated IGHV and del 17p [64]. Combinations with more traditional agents used to treat CLL such as fludarabine and bendamustine have not been as well tolerated, with trials stopped prematurely as a result of the unacceptable toxicities observed [7••, 65]. A unique toxicity in this patient population is the occurrence of “tumour flare”—acute swelling of involved lymph nodes accompanied by an inflammatory response in the overlying skin, rash and fever. This reaction appears to be the result of improved B cell antigen presentation and correlates with clinical response and anti-tumour activity provoked by lenalidomide [66]. Further combinations of lenalidomide with anti-CD20 and anti-CD19 mAbs are being explored in patients with CLL as well as investigation into its use as a maintenance therapy or in those with high-risk but early-stage disease [42].

## Allogeneic Transplantation

The original “adaptive cellular immunotherapy”, allogeneic haematopoietic stem cell transplantation (HSCT), demonstrated the potential that inducing a durable T cell response against a CLL clone can have [67]. Evidence for a graft-versus-leukaemia (GVL) effect was demonstrated by the lower relapse risk observed after chronic graft-versus-host-disease (GVHD), increased relapse seen with T cell depleting strategies [68, 69•] and MRD analyses demonstrating that augmentation of GVL is possible with strategies such as donor lymphocyte infusion (DLI) [70] and HSCT is still regarded as one of the few treatment strategies with the potential to cure CLL.

The indications for allogeneic transplant in patients with CLL established in 2007 by the Society for European Bone Marrow Transplantation (EBMT) suggest its use in high-risk patients with poor prognostic features, “who can expect a significant reduction of life expectancy under alternative therapies” [71]. This translated into an indiction for younger, fitter patients with fludarabine refractory disease, or those with high-risk cytogenetics. Outcomes reported with increasing use of reduced intensity regimens suggest approximate disease-free survival (DFS) ranging between 36 and 43 % and OS rates of 50–63 % at 3–5 years in eligible patients who undergo the procedure [72••].

However, in the current treatment era, with novel agents ibrutinib, idelalisib and BCL2 antagonists demonstrating activity in patients with high-risk CLL [7••, 69•], including those with del 17p and p53 mutations, these guidelines are being questioned and were recently reviewed in the light of this shifting landscape [69•]. Despite improvements in conditioning regimens and supportive care, HSCT remains a procedure with significant associated risk, with non-relapse mortality (NRM) rates in the region of 15–30 % during the first 2 years post-transplant, chiefly the result of infections and GVHD [69•]. Nonetheless, many still consider HSCT as the treatment of choice for eligible patients with high-risk disease [69•].

## Adoptive Cellular Therapy: CAR-T Cells

Hailed as the ultimate weapon in the field of immunotherapy, genetically engineered autologous T cells—or chimeric antigen receptor (CAR) T cells—have been touted as the modern alternative to allogeneic transplant [73]. Bespoke to every patient, native T cells are harvested and engineered ex vivo such that they are redirected to recognize a TSA in conjunction with a co-stimulatory motif. These cells are then expanded and re-infused to generate an adoptive T cell-mediated cytotoxic response—Fig. 1. Attempts to improve in vivo persistence and the cytotoxic capability of CAR T cells have led to the inclusion of either CD137 (4-1BB) or CD28 signaling domains, and these so-called second generation CAR-T cells have improved anti-tumour efficacy.

In the setting of CLL, the conditioning regimen and co-stimulatory motif may be particularly important to ensure expansion and persistence of the CAR-T cells post-transfer. Regulatory T cells (T_regs_) are expanded in CLL patients; thus, it is likely that a conditioning regimen capable of eliminating these and other detrimental subpopulations may be crucial [74, 75]. In addition, the use of the CD137 co-stimulatory domain appears less likely to trigger pro-inflammatory cytokine release (e.g., IL-2 and TNFα) that can also promote the differentiation of T_regs_, and this strategy appears to improve the persistence of transferred CARs in vivo [76].

CD19-directed CAR-T cells have demonstrated impressive clinical results both in acute lymphoblastic leukaemia [77, 78] and an increasing number of patients with CLL [79]. Overall response rates in relapsed and refractory CLL patients approaching 45 % have been reported by the group from the University of Pennsylvania with long-term remissions observed even in patients with bulky disease [72••]. Persistence of CAR-T cells beyond 3 years has also been reported [79, 80]; thus, this therapy may hold promise for long-term disease control. Future approaches that may prove efficacious in using CAR-T cells to treat patients with CLL (apart from optimizing the conditioning regimen and CAR itself) may involve combining CAR-T cells with checkpoint inhibitors, selecting memory T cells to enhance persistence or including transgenes that protect the CARS from an inhibitory microenvironment [72••, 81].

When directly compared to HSCT, CAR-T cells have two key advantages, namely an absent risk of GVHD and lack of requirement for long-term immunosuppression [72••]. The downside is the induction of B cell aplasia since normal CD19-expressing B cells are also eliminated. These patients therefore require long-term immunoglobulin replacement. However, considering the median age of CLL diagnosis lies somewhere between 67 and 72 years of age [7••], many patients with high-risk features are not considered eligible for HSCT due to the significant treatment-related mortality. These patients might, in the future, be candidates for CAR-T cell therapy, although it remains in the early stages of development and technically challenging to deliver outside of specialist centres.

## Conclusion

What is clear from the data outlined above and the rapid development in immunotherapy observed to date is that directing the immune system to target a malignant clone can be a very efficacious strategy. Haemato-oncology has been at the forefront of immunotherapeutic innovation for decades, and CLL is a condition that lends itself extremely well to the investigation of various immune interventions with readily accessible tissue from both malignant and infiltrating immune cells [2••].

A key underpinning feature of the immune system remains that distinct effector subpopulations act in a co-ordinated fashion to exert or amplify the immune response. Thus, it seems likely that a multi-faceted, combination approach is what ultimately will be required to generate maximal benefits from these novel agents. This strategy has already been adopted by trials looking at combinations of anti-CD20 mAbs with anti-PD1 antibodies or lenolidomide, and the number and variety of combinations being tested in the clinic seems likely to continue to expand.

Another potential opportunity for development is in the targeting of early-stage disease, generating an anti-tumour response prior to the development of an immunosuppressive, pro-neoplastic microenvironment. This has been studied in the setting of advanced-stage, asymptomatic, non-bulky follicular lymphoma using single agent rituximab, with significant differences observed in progression-free survival favouring those in the rituximab-treated versus the “watch-and-wait” arm (hazard ratio 0.23, 95 % CI 0.16–0.32, *p* < 0.0001) [82]. A trial involving patients with early-stage, asymptomatic CLL and high-risk cytogenetics (del 17p or 11q) using single agent lenalidomide was also attempted but was unfortunately terminated early due to poor recruitment (NCT01649791). Given what is known about tumour burden augmenting immunotherapeutic resistance through a variety of mechanisms, it would seem likely that exploiting early recognition by the immune system would have “substantial therapeutic impact” [2••].

With the advancement of diagnostic technology, such as next-generation sequencing, it would not be unreasonable to foresee that a more tailored and individual approach may be possible in the future. Molecular signatures from both CLL and immune cells could be profiled and used to identify optimal immunotherapeutic strategies. As in other haematological malignancies, the future for patients with CLL has never been better. As our understanding of the nature of the immune defects and survival pathways in this disease expands, so too does the available therapeutic arsenal. What remains challenging is determining exactly how this vast array of novel immunotherapeutics will fit in to the rapidly changing treatment paradigm.
